# Breast Cancer Is Increased in Women With Primary Ovarian Insufficiency

**DOI:** 10.1210/clinem/dgae480

**Published:** 2024-07-12

**Authors:** Kristina Allen-Brady, Barry Moore, Lauren E Verrilli, Margaret A Alvord, Marina Kern, Nicola Camp, Kristen Kelley, Joseph Letourneau, Lisa Cannon-Albright, Mark Yandell, Erica B Johnstone, Corrine K Welt

**Affiliations:** Division of Epidemiology, Department of Internal Medicine, University of Utah School of Medicine, Salt Lake City, UT 84108, USA; Utah Center for Genetic Discovery, Department of Human Genetics, University of Utah, Salt Lake City, UT 84112, USA; Division of Reproductive Endocrinology and Infertility, Department of Obstetrics and Gynecology, University of Utah School of Medicine, Salt Lake City, UT 84112, USA; Intermountain Healthcare, Murray, UT 84107, USA; Division of Endocrinology, Metabolism and Diabetes, Department of Internal Medicine, University of Utah School of Medicine, Salt Lake City, UT 84112, USA; Division of Endocrinology, Metabolism and Diabetes, Department of Internal Medicine, University of Utah School of Medicine, Salt Lake City, UT 84112, USA; Huntsman Cancer Institute and Department of Internal Medicine, University of Utah School of Medicine, Salt Lake City, UT 84132, USA; Huntsman Cancer Institute and Department of Internal Medicine, University of Utah School of Medicine, Salt Lake City, UT 84132, USA; Division of Reproductive Endocrinology and Infertility, Department of Obstetrics and Gynecology, University of Utah School of Medicine, Salt Lake City, UT 84112, USA; Division of Epidemiology, Department of Internal Medicine, University of Utah School of Medicine, Salt Lake City, UT 84108, USA; Utah Center for Genetic Discovery, Department of Human Genetics, University of Utah, Salt Lake City, UT 84112, USA; Division of Reproductive Endocrinology and Infertility, Department of Obstetrics and Gynecology, University of Utah School of Medicine, Salt Lake City, UT 84112, USA; Division of Endocrinology, Metabolism and Diabetes, Department of Internal Medicine, University of Utah School of Medicine, Salt Lake City, UT 84112, USA

**Keywords:** prostate cancer, menopause, genetics, ovarian cancer

## Abstract

**Context:**

DNA damage/repair gene variants are associated with both primary ovarian insufficiency (POI) and cancer risk.

**Objective:**

We hypothesized that a subset of women with POI and family members would have increased risk for cancer.

**Design:**

Case-control population-based study using records from 1995 to 2022.

**Setting:**

Two major Utah academic health care systems serving 85% of the state.

**Subjects:**

Women with POI (n = 613) were identified using International Classification of Diseases codes and reviewed for accuracy. Relatives were linked using the Utah Population Database.

**Intervention:**

Cancer diagnoses were identified using the Utah Cancer Registry.

**Main Outcome Measures:**

The relative risk of cancer in women with POI and relatives was estimated by comparison to population rates. Whole genome sequencing was performed on a subset of women.

**Results:**

Breast cancer was increased in women with POI (OR, 2.20; 95% CI, 1.30-3.47; *P* = .0023) and there was a nominally significant increase in ovarian cancer. Probands with POI were 36.5 ± 4.3 years and 59.5 ± 12.7 years when diagnosed with POI and cancer, respectively. Causal and candidate gene variants for cancer and POI were identified. Among second-degree relatives of these women, there was an increased risk of breast (OR, 1.28; 95% CI, 1.08-1.52; *P* = .0078) and colon cancer (OR, 1.50; 95% CI, 1.14-1.94; *P* = .0036). Prostate cancer was increased in first- (OR, 1.64; 95% CI, 1.18-2.23; *P* = .0026), second- (OR, 1.54; 95% CI, 1.32-1.79; *P* < .001), and third-degree relatives (OR, 1.33; 95% CI, 1.20-1.48; *P* < .001).

**Conclusion:**

Data suggest common genetic risk for POI and reproductive cancers. Tools are needed to predict cancer risk in women with POI and potentially to counsel about risks of hormone replacement therapy.

Primary ovarian insufficiency (POI) is defined as primary hypogonadism in women before the age of 40 years (1). Autoimmune causes, *FMR1* premutations, and X chromosome deletions and translocations account for a majority of POI. In the remaining cases, next-generation sequencing demonstrates a candidate or causal gene in up to 43% of women (2). Approximately 50% of the deleterious gene variants causing POI were found in genes involved in meiosis, DNA damage repair and transcription, and translation fidelity (2). Mutations in these genes could predispose to cancer risk in women with POI (3, 4).

The association between POI and cancer was recognized in families with recessive variants in *MCM9* or *BRCA2* causing POI (5, 6). In those families, colon, metastatic cervical cancer, and acute myelocytic leukemia occurred in the same girls at early ages (5, 6). In addition, there are known early cancer syndromes associated with POI: Bloom (*BLM*), Nijmegen breakage (*NBN*), ataxia telangiectasia mutated (*ATM*), and Werner (*WRN*) syndromes (1, 7-10). However, the relationship between POI and personal and familial cancer has not been examined outside of these families with recessive and syndromic presentations.

Infertility, in general, has been associated with cancer risk in some studies. One prospective study demonstrated that infertility is associated with an increased risk of postmenopausal breast cancer (11). The relationship was stronger for primary infertility and earlier age at infertility diagnosis, which could be related to ovarian insufficiency, although that specificity was not available (11). In contrast, genome-wide variants associated with later age at natural menopause increased risk for endometrial and breast cancer (12). The balance of potential genetic risk from earlier menopause and the prolonged hormone stimulation of later menopause needs to be separated.

We hypothesized that women with POI would have increased risk of reproductive cancers, including breast, ovarian, and endometrial cancers. We also examined colon cancer based on its higher prevalence in carriers of cancer risk genes and decreased incidence with hormone replacement therapy (13, 14). Based on the familiality of POI (15), we also hypothesized that family members of women with POI would have increased reproductive and hormonally sensitive cancers, including prostate and testicular cancer in men. Studying women with POI, their medication history, and their relatives using the Utah Population Database (UPDB) and electronic medical records (EMRs), we were able to demonstrate increased reproductive cancer risk in women with POI and their families.

## Materials and Methods

### POI Cases

We identified women aged ≤40 years with POI using EMRs from 1995 to 2022 at the University of Utah Health and Intermountain Healthcare, which together serve 85% of all residents in Utah (15). Cases of POI were ascertained through International Classification of Diseases (ICD) codes: ICD-9 (256.3, 256.31, 256.39) and ICD-10 codes (E28.3, E28.31, E28.39, E28.310, E28.319), EMR notes indicating POI diagnoses, and/or laboratory values (elevated FSH > 20 IU/L or anti-Mullerian hormone <0.08 ng/mL in a woman younger than age 40 years at the time of the laboratory draw). Subjects were excluded for hysterectomy, oophorectomy, endometriosis with pelvic surgery, pelvic radiation, or chemotherapy before the diagnosis of POI. We also excluded subjects with Turner syndrome (ICD-9 758.6 and ICD-10 Q96). In addition, women with early menopause at age <45 and >40 years were identified using the same inclusion and exclusion criteria as for POI.

The initial list of identified POI and early menopause cases were verified through individual chart review by a medical or reproductive endocrinologist (C.K.W. or L.E.V.) for inclusion. Validation factors included FSH and anti-Mullerian hormone levels, type of physician making the diagnosis, and POI symptoms and signs, as outlined previously (15).

### Pedigree Creation

We used the UPDB to link genealogy information to medical record information and data from the Utah Cancer Registry (UCR) for women with POI (16). Medical record numbers for women with POI were converted to UPDB identification numbers by an independent oversight group under the requirements for use. Subsequently, UPDB identifications were linked to genealogy contained within the UPDB. For the familiality analysis, POI probands were required to have at least 3 generations of genealogy information available (proband, both parents, and all grandparents, or 6 or more ancestors). Three generations increase the likelihood that the subjects resided in Utah for many generations and that complete family data, health care data, and cancer data are available for any woman.

### Ethics

The University of Utah and Intermountain Healthcare institutional review boards and the Resource for Genetic and Epidemiologic Research, overseers of UPDB data, approved this study. A waiver of consent was obtained for the familiality study and chart review. Signed consent was obtained for the subset of subjects who provided DNA.

### Identification of Cancer Cases

The records of probands with a verified POI diagnosis in the UPDB, and their family members were screened for cancer diagnoses using the existing linkage between the UPDB and the UCR. National Cancer Institute Surveillance Epidemiology and End Result registry codes for breast (26000), corpus uteri (27020-30), ovary (27040), prostate (28010), testis (28020), and cecum/colon (21041-46) cancers were identified for each subject.

### Cancer Relative Risk Analysis

We estimated the relative risk (RR) of individual cancers in women with POI and their first-, second-, and third-degree relatives. Of note, first-degree relatives include parents, siblings, and children of cases. Second-degree relatives include grandparents, aunts/uncles, nieces/nephews, half-siblings, and grandchildren. Third-degree relatives include great-grandparents, great-grandchildren, and first cousins. The relative risk of a disease estimates how likely an individual is to develop a disease if he or she has POI or is a relative of a woman with POI. Relative risk was estimated as the ratio of the observed number of cases of a specific type of cancer for a woman with POI or a specific relative type (eg, first-degree relatives) compared to the expected number of cancer cases for a woman or a specific relative type. The expected number of cancer cases was calculated based on population rates calculated for each 5-year birth cohort represented by the POI cases or relatives and birthplace (Utah or outside of Utah) within the University of Utah Health and Intermountain Healthcare. The rate was defined by the total number of cancer cases within a cohort divided by the total cohort size. The number of expected cases was calculated as the sum of each cohort-specific cancer risk for each individual in a set of relatives of a specific type (eg, first-degree relatives). Approximate 95% CIs and exact hypothesis tests of the null hypothesis (RR = 1.0) were constructed assuming that the number of cancer cases found among the relatives follows a Poisson distribution. Many RR sampling schemes can lead to bias or inflated estimates. The studies we performed with the UPDB are population-based, reducing the risk for sampling bias that results from proband identification and oversampling from pedigrees with multiple affected members. We corrected for multiple testing for 4 cancer types in women (*P* < .05/4 = 0.012) and 3 cancer types in men (*P* < .05/3 = 0.017).

### Identification of High-Risk Cancer Pedigrees

High-risk cancer pedigrees among POI subjects' pedigrees were identified (15). Briefly, high-risk cancer pedigrees were defined as those pedigrees with a significant excess number of cancer cases among ancestors. We compared the observed number of cancer cases in the pedigrees of POI probands to the expected number of cancer cases for all ancestors based on population rates of cancer using population controls matched for 5-year birth cohort, sex, and birthplace (Utah or not). For every member in each pedigree, the expected number of cancer cases was calculated as the cohort specific cancer rate, summed across all pedigree members. Pedigrees with a statistical excess (*P* < .05) number of observed cancer cases compared to the expected number of cancer cases were considered to be high risk.

### Genetic Analysis

DNA samples were extracted (Qiagen) and subjected to whole genome sequencing using the Novaseq X (Illumina). Alignment and variant calling were performed by the Utah Center for Genetic Discovery pipeline using the Sentieon software package (https://www.sentieon.com) (17). Reads were aligned to the human reference build GRCh38/hg38 using BWA-MEM (Burrows–Wheeler Aligner). SAMBLASTER was used to mark duplicate reads and deduplicate aligned BAM files. Aligned BAM files underwent INDEL realignment and base recalibration using Realigner and QualCal algorithms from the Sentieon software package3 to produce polished BAM files. Each polished BAM file was processed using the Sentieon's Haplotyper algorithm to produce gVCF files (18). Sample gVCF files were combined and jointly genotyped with 728 samples comprising the 1000 Genomes Project (CEU) samples and samples unrelated to reproduction or cancer phenotypes to produce a multisample VCF file. To produce the final VCF variant quality scores, VCF files were recalibrated using Sentieon's VarCal algorithm to estimate the accuracy of variant calls and reduce potential false-positive calls. Quality control algorithms were applied to sequence reads (Fastq files), aligned reads (BAM files), and variants (VCF files) (19). Fastp was used to evaluate read quality, read duplication rate, presence of adapter, and overrepresented sequences in Fastq files (20). Indexcov was used to estimate depth and coverage of aligned sequence data using BAM indexes. Further alignment quality metrics were calculated on BAM files with samtools stats. Variant quality metrics were calculated by running bcftools stats (21-23). The overall quality of VCF call sets were evaluated using Peddy to confirm sex, relatedness, heterozygosity, and ancestry of each individual and identifying potential sample-level data quality issues (24).

Genetic variants were prioritized using GEM (25). GEM generates a Bayes factor-based score that calculates the degree of support for and against a given model (a gene allele is pathogenic vs benign) considering multiple lines of evidence from: Variant Annotation, Analysis, and Search Tool, Variant Annotation, Analysis, and Search Tool Variant Prioritizer, Phenotype Driven Variant Ontological Re-ranking tool, mode of inheritance for disease genes from Online Mendelian Inheritance in Man, pathogenicity of variants, population-specific allele frequencies (gnomAD), quality of variants and the overall genome, and quality of the genomic location (gnomAD) (26-29). Using these data, GEM identifies potentially pathogenic genotypes and evaluates support for their association with disease; POI and neoplasm for the current data. Gene variants were considered candidates if they had a GEM score ≥ 1 (strong support for the model of pathogenicity), together with genes that had a GEM score ≥ 0.69 (substantial support for the model of pathogenicity), and a Phenotype Driven Variant Ontological Re-ranking tool Bayes factor ≥ 0.9 (genes with a strong association with POI in conjunction with additional phenotypes identified by associated Human Phenotype Onotolgy terms) (27).

## Results

We identified 613 women diagnosed with POI from the University of Utah and Intermountain Healthcare. The age of the women at the time of the POI diagnosis was 32.7 ± 7.4 years (range, 12-40) and at the time of the study was 48.3 ± 11.8 years (19-93). These women were predominantly of self-reported White (78.8%), White Hispanic (10.4%), Asian (1.6%), and multiple races (3.7), with the rest Native American, Pacific Islander, Black, and unknown. Of the 613 total, 416 women with POI also had 3 generations of genealogical data available in the UPDB. At the time of the study, their age was 47.3 ± 11.9 years. These 416 women had 2405 first-degree relatives, 6798 second-degree relatives, and 17 666 third-degree relatives.

We found an increased risk of breast cancer in probands, with 18 women affected (Table 1). Probands were aged 36.5 ± 4.3 years when diagnosed with POI and aged 59.5 ± 12.7 years (range, 43–80 years) at the time of breast cancer diagnosis and their body mass index was 28.6 ± 6.4 kg/m^2^ (range, 19.6-47.5 kg/m^2^). Of the 13 breast cancer cases with pathology data available, most were invasive ductal (93%) with the majority ER+ (77%), half PR+ (54%), and a minority HER2+ (17%). Genetic testing had been performed on <10 women and there were no pathogenic mutations identified, although 1 variant of uncertain significance was found in *BRIP1* (c.415T>G, p.Ser139Ala). Five women never took hormone replacement therapy (28%); 3 took hormonal contraception for 4, 5, and 10 years before menopause (17%); and 3 had no information (17%). The rest of the subjects took some form of hormone replacement therapy after the diagnosis of POI. Four took hormone replacement for <1 to 4 years and 1 for 10 years but stopped before age 50 years (28%). Only 2 women (11%) took hormone replacement therapy after age 50 years and had the latest ages at cancer diagnosis, after age 75 years. Only 2 of the women had no children. There were 8 (44%) past or current smokers. Sixteen of the women had a family history of cancer (89%), with renal, melanoma, hematologic, lung, bladder, sarcoma, and pancreatic cancers in addition to those examined in the current study. When a Gail Model Score was derived for the women at the age of their breast cancer diagnosis, n = 9 had a lower risk score for 5-year and lifetime risk of breast cancer compared to the US population mainly related to first birth before age 25 years, whereas n = 4 had a higher risk score mainly related to a first-degree relative with breast cancer (including the woman with the *BRIP1* variant of uncertain significance), 1 was similar and n = 4 could not be calculated.

**Table 1.** Cancer identified in women with POI

Cancer type	Observed #	Expected #	Relative risk (95% CI)	*P* value
Probands (n = 613)				
Breast	18	8.19	2.20 (1.30-3.47)	.0023*
Ovarian	≤10	0.82	3.67 (1.00-10.71)	.050
Uterine	≤10	1.42	1.40 (0.17-5.07)	.66

The observed number of subjects with cancer, expected number based on population rates, and the relative risk. Breast and ovarian cancer relative risk is increased in women with POI (*P*S ≤ .05), although ovarian cancer risk was not significant after multiple testing. Observed numbers were listed as ≤10 based on the regulations of the UPDB oversight board, the Resource for Genetic and Epidemiologic Research to protect the identify of small numbers of patients. There was no colon cancer found in these women.

Abbreviations: POI, primary ovarian insufficiency; UPDB, Utah Population Database.

^*^Bonferroni multiple testing correction *P* < .012.

In addition to breast cancer, there was a borderline increased risk of ovarian cancer, papillary serous, in POI probands (Table 1). There was no increased risk of endometrial cancer and there were no cases of colon cancer.

For the 6 subjects with DNA that underwent whole genome sequencing, there were candidate genes identified (Table 2 and Supplementary Information) (30). Two of the women had frameshift variants: 1 in *RAD51D* (p.His250ThrfsTer2) and 1 in *MORC2* (p.Leu67PhefsTer3), and 1 woman had a stop gain and missense variant in *FANCM* (p.Gln1701Ter and p.Gly546Ser), all considered pathogenic, whereas the variants in the rest of the subjects were missense variants in *ERCC6* (p.Pro591Leu), *FANCD2* (p.Arg1299Cys), and *MCM8* (p.Ile557Val) of uncertain significance or with conflicting interpretations (29).

**Table 2.** Candidate gene variants in women with POI and a subsequent cancer diagnosis

POI age (y)	Gene	Chr: location	Protein change	Consequence (*)	Cancer diagnosis	CA age (y)	Family history of cancer
36	*RAD51D*	17:35101355_TG	p.His250ThrfsTer2	Frameshift*	High-grade serous Fallopian tube	65	MA, MGM ovarian, Glioblastoma and glioma
35	*MORC2*	22:30950402 C>CA	p.Leu67PhefsTer3	Frameshift*	Invasive ductal breast	46	None
30	*FANCM*	14:45189123 C>T	p.Gln1701Ter	Stop gain*	Invasive ductal breast ER−/PR−HER2−	43	M, MA breast, M, F, S melanoma, MU prostate, MGF lung, MGM multiple myeloma, MGM, MA endometrial
		14:45164413 G>A	p.Gly546Ser	Missense			
40	*ERCC6*	10:49493166 G>A	p.Pro591Leu	Missense	Invasive ductal breast ER+/PR+HER2−	67	M melanoma, MU melanoma, B melanoma, MGF, M1C pancreatic
40	*FANCD2*	3:10094295 C>T	p.Arg1299Cys	Missense	Invasive ductal breast ER+/PR+HER2−	70	S colon, PGM ovarian
39	*MCM8*	20:5983101 A>G	p.Ile557Val	Missense	Invasive ductal breast ER−/PR−HER2−	62	M breast

Age at POI, POI/cancer presumed causal gene, variant location, protein change, effect on the protein, along with CA diagnosis and age and family history of cancer. All variants were heterozygotes. These variants are novel or extremely rare in gnomAD (minor allele frequency <.001) (28).

Abbreviations: 1C, first cousin; A, aunt; B, brother; CA, cancer; F, father; G, grand; M, mother/maternal; NA, not available; P, paternal; POI, primary ovarian insufficiency; S, sister; U, uncle.

^*^Pathogenic or likely pathogenic (29).

When we included women with early menopause (n = 165) to the group of women with POI (total n = 778), the risk of breast cancer remained significant with 23 observed versus 12.15 expected breast cancer cases (OR, 1.89; 95% CI, 1.20-2.84; *P* = .0056). Ovarian cancer was also nominally significant (OR, 3.38; 95% CI, 1.15-8.65; *P* = .032). There were ≤10 cases of endometrial and colon cancer in the early menopause group, and the RR was not greater than expected based on population rates. The Gail Model Score in the early menopause subjects was lower risk for n = 1, similar for n = 1, higher for n = 2, and unable to be calculated in n = 1, for reasons similar to those in the POI subjects.

In women with POI and 3 generations of family members, the increased relative risk of breast cancer remained significant despite smaller numbers (Table 3). There was an increased risk of breast cancer and colon cancer in second-degree relatives, but not first-degree relatives (Table 3). The relative risk of prostate cancer was increased in all relative groups (Table 3).

**Table 3.** Cancer in women with POI and their first-, second-, and third-degree relatives

Relative type	Cancer type	Observed	Expected	RR (95% CI)	*P* value
Self (n = 416)	Breast	13	5.30	2.45 (1.31-4.19)	.0033*
	Ovarian	≤10	0.57	3.50 (0.42-12.66)	.11
	Colon/colorectal	≤10	0.6	0 (0-6.13)	1.00
	Uterine	≤10	0.93	2.16 (0.26-7.80)	.24
First degree (n = 1236 males; n = 1169 females)	Breast	32	26.42	1.21 (0.833-1.71)	.28
	Ovarian	≤10	2.94	1.70 (0.55-3.97)	.23
	Colon/colorectal	≤10	7.41	1.08 (0.47-2.13)	.71
	Uterine	≤10	5.45	0.37 (0.04-1.33)	.19
	Prostate	41	24.96	1.64 (1.18-2.23)	.0026*
	Testicular	≤10	2.21	1.36 (0.28-3.97)	.49
Second degree (n = 3458 males; n = 3340 females)	Breast	127	99.58	1.28 (1.06, 1.52)	.0078*
	Ovarian	12	12.39	0.97 (0.50-1.69)	1.00
	Colon/colorectal	58	38.71	1.50 (1.14-1.94)	.0036*
	Uterine	25	23.53	1.06 (0.69-1.57)	.76
	Prostate	174	112.72	1.54 (1.32-1.79)	< .001*
	Testicular	≤10	4.19	0.95 (0.26, 2.44)	1.00
Third degree (n = 9009 males; n = 8657 females)	Breast	230	227.36	1.01 (0.89-1.15)	.84
	Ovarian	37	30.76	1.20 (0.85-1.66)	.24
	Colon/colorectal	123	111.53	1.11 (0.92-1.33)	.24
	Uterine	49	58.24	0.84 (0.62-1.11)	.26
	Prostate	364	272.94	1.33 (1.20-1.48)	< .001*
	Testicular	11	10.23	1.08 (0.54-1.92)	.75

Abbreviations: POI, primary ovarian insufficiency; RR, relative risk.

^*^Bonferroni multiple testing correction *P* < .012 for women and *P* < .017 for men.

**Figure 1.**
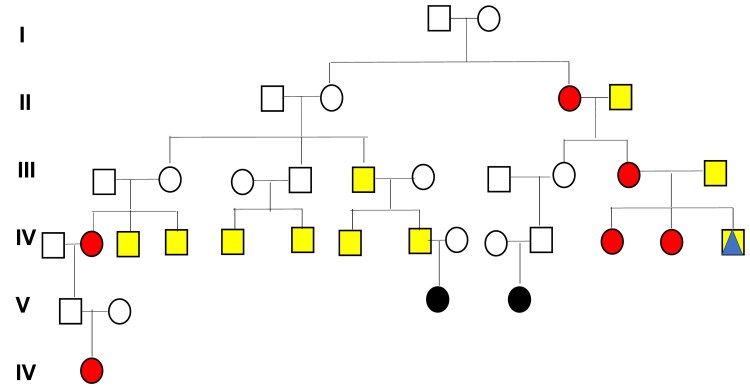
High-risk prostate and breast cancer pedigree. Circles are females and squares are males. Black circles are females affected with primary ovarian insufficiency (POI). Red/filled circles are women with breast cancer and yellow/filled squares are men with prostate cancer. There is 1 case of colon cancer indicated by a triangle. Note: The pedigree has been trimmed to ensure confidentiality.

There were 27 POI pedigrees that could be considered high risk for reproductive cancer diagnoses (Table 4). Two high-risk pedigrees demonstrated an excess of both breast and prostate cancer (Fig. 1): 1 had an excess of ovary and prostate and another had an excess of colorectal and prostate cancer.

**Table 4.** Increased cancer risk in primary ovarian insufficiency pedigrees

Cancer type	Pedigree	Observed	Expected	*P* value
Breast	1	70	46.79	.00091
	2	33	19.48	.0032
	3	58	40.74	.0063
	4	78	61.45	.024
	5	102	83.87	.030
	6	43	32.58	.046
Colorectal	7	48	28.80	.00066
	8	23	11.72	.0023
	9	14	6.92	.012
	10	27	17.02	.015
	11	41	29.57	.027
	12	14	8.38	.047
Ovary	13	12	3.17	.00012
	14	≤10	3.46	.0031
	15	13	7.1	.030
	16	≤10	2.51	.043
Prostate	17	143	110.24	.0016
	18	30	20.04	.022
	19	176	151.57	.028
	20	58	45.85	.047
Endometrial	21	≤10	2.19	.025
Testis	22	≤10	2.56	.046
Ovary/prostate	23	≤10/32	2.55/23.29	<.05
Colon/prostate	24	11/24	5.11/15.42	<.03
Breast/prostate	25	44/42	30.61/27	<.02
	26	≤10/≤10	1.51/1.90	<.005
Breast/ovary	27	28/≤10	19.47/2.35	<.05

Four pedigrees demonstrated an excess of more than 1 type of cancer.

## Discussion

We demonstrate an increase in breast cancer risk compared to population rates in women with POI. There was also a borderline increased risk of ovarian cancer in women with POI and early menopause. A subset of the women with available DNA carried rare gene variants associated with both POI and cancer risk. In relatives of women with POI, there was an increased risk for breast and colon cancer in second-degree relatives and prostate cancer in all relative types examined. These findings suggest common risk for POI and reproductive or hormonally sensitive cancers.

In a subset of the women with DNA available, we identified gene variants that could predispose to both POI and cancer in women with early-onset or high-grade cancer. These include rare, deleterious, heterozygous variants in genes that repair DNA after recombination during meiosis I in oocytes and in cells with DNA damage (2). One subject with a rare fallopian tube cancer and family history of ovarian cancer had a frameshift mutation in *RAD51D,* which has been associated with cancer risk (31). Its interaction BLM also suggests that it may play a role in ovarian insufficiency, similar to other *RAD51* family genes (32, 33). We identified presumed compound heterozygous *FANCM* variants in a woman with triple-negative breast cancer. The homozygous stop gain p.Gln1701Ter has been associated with POI onset before age 25 years (34). One copy increases the risk of breast cancer approximately 2-fold in heterozygotes (35, 36). The missense variant, located in the helicase C-terminal domain, is classified as pathogenic and increases risk in triple-negative, familial breast cancer (37). Finally, *MORC2* has been identified as an oncogene and deletion in mouse models results in infertility (38, 39).

Additional variants were identified in women with age at menopause closer to age 40 years, and with ER+/PR+ breast cancers. We identified a heterozygous variant in *FANCD2*, a gene in which we previously found a protein truncating variant in a woman with POI (2). The location changing a p.Arg1299Cys would disrupt the helix structure (40). However, it is unknown whether a heterozygous variant could increase cancer risk (36). A stop gain variant in *MCM8* has previously been associated with earlier age at menopause in a carrier mother at the age of 28 years (41). The *ERCC6* gene has been implicated in autosomal dominant POI and in cancer (7, 42).

Prostate cancer was the most common cancer in families of women with POI. Some of the same genes that increase risk for breast cancer are also associated with prostate cancer. *BRCA2* and *BRCA1* variants are associated with hereditary predisposition to prostate cancer (43). An increased risk of prostate cancer for carriers of mutations in *ATM* has also been reported, and further investigation is needed to establish the level of increased risk for carriers of mutations in the partner and localizer of *BRCA2* (*PALB2*), nibrin (*NBN*), and more recently implicated DNA repair genes, such as recombination protein A (*RAD*) *51*, Fanconi anemia complementation group A (*FANCA*) and *BRIP1* (43-49). Additionally, variants in genes associated with Lynch syndrome, *MSH2* and *MSH6*, have been implicated (43-49). These genes are also important for DNA damage repair and variants may be the common denominator between POI in women and prostate cancer in male relatives.

There was an increased risk for breast and colon cancer only in second-degree relatives. More first-degree relatives (51%) were younger than age 50 years compared to second-degree relatives (39%) and only 19% of first-degree relatives were older than age 65 years, perhaps explaining the lack of association with breast and colon cancer in that group. If a common genetic background was responsible for these cancers, breast cancer risk genes may be implicated, similar for risk in probands (50). Familial colon cancer, like prostate cancer, has a significant overlap with breast cancer risk (51). The DNA damage repair genes implicated in colon cancer are the same as those involved in risk for breast and prostate cancer. In particular, *MCM9* recessive mutations cause both POI and colorectal cancer (52). In addition to DNA damage repair genes, tumor suppressor genes such as *APC* and *PTEN,* and TGF-β genes such as *SMAD4* and *BMPR1A* are also associated with colorectal risk and some have been implicated in POI (51, 53, 54). Thus, the genetics in relatives with colon and breast cancer may also overlap with POI risk genes in these families.

In contrast to the current data, genome-wide association studies suggest that genetic risk for later age at menopause is associated with breast cancer (12). These relationships are likely because of longer hormonal exposure (55). Only 2 women used hormone replacement therapy beyond age 50 years in the current study and they had the latest breast cancer diagnoses, after age 75 years. The rest did not take hormone replacement, including the women with earliest age at breast cancer diagnosis, or took it only until age 50 years, as recommended by experts (56, 57). These data suggest that genetic risk and not hormone use drives the increased risk for breast cancer in younger women with POI, whereas breast cancer associated with hormone replacement therapy may be less specific for POI. It is currently recommended that hormone replacement therapy (estradiol and a progestin) be prescribed for women with POI until the average age at menopause, approximately age 50 to 52 years (56, 57). The only exceptions would be for women with a family history of breast cancer, in whom it is suggested to stop hormone replacement at age 45 years (56). The current data suggest that genetic testing for breast cancer risk genes in women with a family history of cancer would be appropriate and the risks and benefits of hormone replacement therapy considered in the context of these genetic results.

Decreased parity and later age at first birth are associated with breast cancer risk (58). Despite the POI diagnosis, there were only 2 women without children, suggesting nulliparity did not play a significant role in breast cancer in these women. We previously demonstrated that fewer women with POI have at least 1 child (54% vs 68%), but those who did have children had only 1 less child than population rates (59). The age at the birth of the first and last child was not different in women with POI compared to the population rates in Utah (59). However, the Utah population had a very high birth rate before 2010, including among 15- to 24-year olds, although it has dropped in recent years (60). Utah also has a relatively low prevalence of breast cancer compared to other states (61). Therefore, the protective aspect of parity generally decreased the calculated breast cancer risk and lower state rates of breast cancer may mean that the estimated risk in the current study is conservative.

It is of interest that the majority of women with POI who developed breast or ovarian cancer were age 30 years or older at the time of their final menses. Further, the relative risk of breast and ovarian cancer increased with the addition of women who had early menopause, before age 45 years. Highly penetrant cancer syndromes are often recessive in inheritance and present with very early-onset cancers and early POI (6, 8-10, 52). Although we have not yet examined cancers other than reproductive-related cancers, the data suggest that cancer risk is more likely caused by a heterozygous, deleterious variant or multiple deleterious or common variants as typical for a complex disorder with later presentation and variable penetrance (2). It is possible that ovarian function will provide a window into the functional effect of the genetic variants that may increase cancer risk but are difficult to interpret. For example, if a risk variant for cancer results in POI or early menopause, perhaps it has a greater in vivo consequence. Therefore, POI could provide a readout of cancer risk through menopause age in combination with genetic risk and hormone exposure.

The strength of the study is the careful case definition of POI at a population level validated with chart review, the extensive genealogy database, and the UCR to confirm cancer in the population. Limitations include elimination of women with very early cancer diagnoses requiring radiation or chemotherapy who might have experienced POI at a later age, but who are excluded from the cohort. We are limited by a largely northern European population, although 10.4% of these women had Hispanic ethnicity (62), but that may not have generalizable results for other races and ethnicities. Finally, we will need genetics to determine whether these family relationships represent true shared genetic risk for these reproductive cancers.

Clinicians need tools to predict cancer risk and comorbid disease in women with POI to adequately counsel about future health in light of our findings. Our data suggest that a subset of women with POI need counseling regarding future cancer risk. Recommendations will likely depend on underlying family history and genetic risk. Further, current practice recommending hormone replacement therapy until the average age at menopause may need to be reconsidered for a subset of women with POI and early menopause who have POI caused by potential cancer risk genes.

## Data Availability

The data supporting the current study have not been deposited in a public repository because they require institutional review board approval and Utah Resource for Genetic and Epidemiology Research approval but are available from the corresponding author on request.

## Abbreviations

EMR: electronic medical record
ICD: International Classification of Diseases
POI: primary ovarian insufficiency
RR: relative risk
UCR: Utah Cancer Registry
UPDB: Utah Population Database
